# Microflow photochemistry: UVC-induced [2 + 2]-photoadditions to furanone in a microcapillary reactor

**DOI:** 10.3762/bjoc.9.237

**Published:** 2013-10-04

**Authors:** Sylvestre Bachollet, Kimitada Terao, Shin Aida, Yasuhiro Nishiyama, Kiyomi Kakiuchi, Michael Oelgemöller

**Affiliations:** 1 James Cook University, School of Pharmacy and Molecular Sciences, Townsville, QLD 4811, Australia; 2 Graduate School of Materials Science, Nara Institute of Science and Technology (NAIST), 8916-5, Takayama-cho, Ikoma, Nara 630-0101, Japan

**Keywords:** cycloaddition, cyclobutane, flow chemistry, furanone, microflow chemistry, photochemistry

## Abstract

[2 + 2]-Cycloadditions of cyclopentene and 2,3-dimethylbut-2-ene to furanone were investigated under continuous-flow conditions. Irradiations were conducted in a FEP-microcapillary module which was placed in a Rayonet chamber photoreactor equipped with low wattage UVC-lamps. Conversion rates and isolated yields were compared to analogue batch reactions in a quartz test tube. In all cases examined, the microcapillary reactor furnished faster conversions and improved product qualities.

## Introduction

**Scheme 1:**
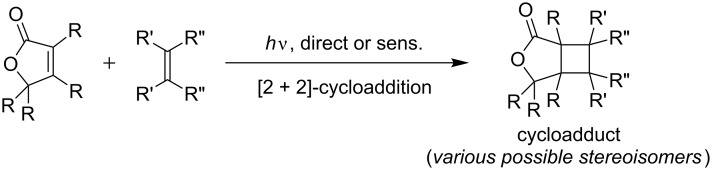
General [2 + 2]-cycloaddition of furanones with alkenes.

Continuous-flow chemistry has recently emerged as a new methodology in organic chemistry [1–4]. The combination of microstructured dimensions and flow operations has also proven advantageous for photochemical applications [5–9]. The narrow reaction channels guarantee efficient penetration of light and yield improved photonic efficiencies [10–11]. Likewise, the removal of the photoproducts from the irradiated area minimizes the risk of photodecompositions or secondary photoreactions [12–13]. Of the many photochemical reactions [14–16], [2 + 2]-photocycloadditions are especially interesting transformations since they allow for the construction of cyclobutanes under mild conditions [17–19]. A number of intra- as well as intermolecular [2 + 2]-photocycloadditions have consequently been described under continuous-flow conditions [20–22]. In an extension of our previous work on furanones [10,23], we have now studied intermolecular photoadditions of alkenes to these compounds [24–25]. Direct and sensitized protocols have both been described (Scheme 1). Sensitized additions allow for irradiations in the UVB range [26–28], whereas direct irradiations require UVC light instead [29–31].

## Results and Discussion

### Experimental setups

**Figure 1:**
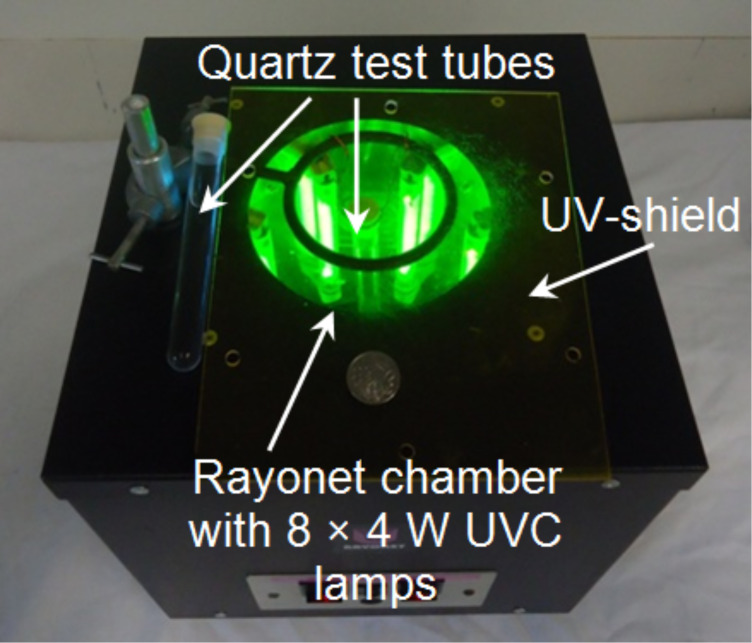
Rayonet chamber reactor (RMR-600; Southern New England) with quartz test tubes. A 10 AU-cent coin is shown for comparison.

The reaction setup for batch irradiations is shown in Figure 1. A commercially available Rayonet chamber reactor (RMR-600; Southern New England) equipped with eight 4 W UVC lamps (λ = 254 nm; arc length: 7.6 cm) in a circular arrangement was chosen. The central chamber was manufactured from highly reflective aluminum and was approximately 23 cm deep and 18 cm in diameter. The reactor is cooled by an integrated fan and temperatures inside the chamber did not exceed 30 °C. Quartz test tubes (length: 12.7 cm; outer/inner diameter: 15/13 mm; filling volume: 10 mL; filling height: 7.6 cm), sealed with a precision seal septum, were used as reaction vessels and were hung into the centre of the chamber. After a preset irradiation time, the reaction mixture was concentrated to dryness and the crude product was analyzed by ^1^H NMR spectroscopy. Conversions were determined by comparing the integration areas of selected signals from the starting furanone and the cycloaddition product. In selected cases, the pure products were isolated by column chromatography for characterization purposes and yield determination.

**Figure 2:**
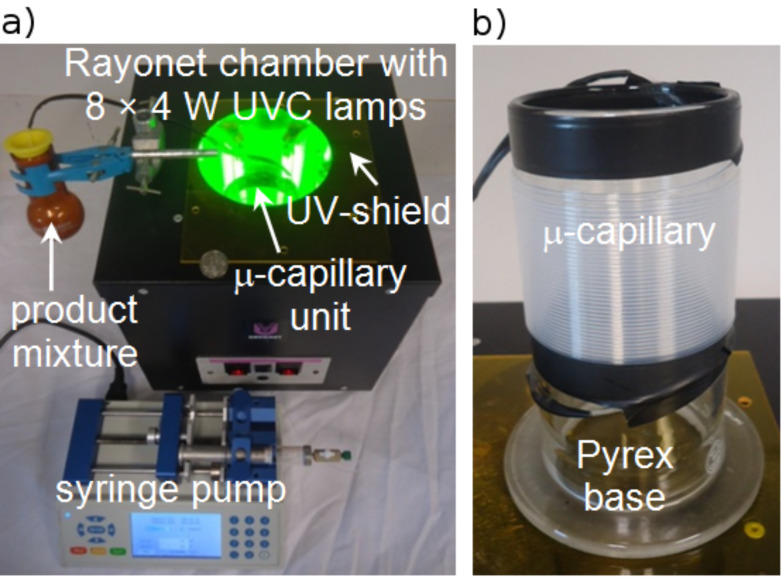
Microcapillary reactor. (a) Setup with inserted μ-capillary unit. A 10 AU-cent coin is shown for comparison. (b) μ-Capillary unit.

The microcapillary reactor setup is shown in Figure 2. UV-transparent fluorinated ethylene propylene copolymer capillary (FEP; outer/inner diameter: 1.6/0.8 mm) was tightly wrapped around a Pyrex glass cylinder (λ ≥ 300 nm; outer diameter: 8.5 cm). A total of 10 m of the capillary covered the cylinder body (windings: 38; coverage: 6 cm; internal volume: 5 mL). This microcapillary unit was placed in the centre of the Rayonet chamber reactor. The non-exposed ends of the capillary (approximately 50 cm each) were covered with black heat-shrink tubing. The inlet was connected to a shut-valve attached to a 10 mL syringe, whereas the outlet was inserted into an amber round-bottom flask outside the chamber reactor. The reaction mixture was loaded into the syringe, degassed with nitrogen, pumped through the microreactor at a given flow rate and collected in an amber flask.

### Irradiation conditions and light penetration

Model irradiations using furanone **1** and cyclopentene (**2**) in acetonitrile were performed under batch conditions to establish the most suitable reaction conditions (Scheme 1; R = R′ = H, R″ = –(CH_2_)_3_–). Upon direct irradiation with UVC light in a quartz tube for 5 h, almost complete conversion of **1** of 95% was achieved. Solely the *cis-anti-cis* isomer of **3** was obtained and was isolated in a yield of 67% after column chromatography, compared to 36% after distillation as reported in the literature [31]. In contrast, sensitized conditions (5 vol % of acetone and irradiation with UVB light) gave an incomplete conversion of approximately 60%. A complex mixture of various stereoisomers of **3** and several unknown byproducts was obtained, which could not be separated satisfactory. Direct irradiation conditions were thus chosen for all further investigations. However, higher cycloalkenes (cyclohexene and *cis*-cyclooctene) gave stereoisomeric mixtures even under these direct irradiation conditions.

Microflow photochemical syntheses with UVC light are rare. Jamison and coworkers have recently used custom-made quartz coils [32–33], however, these are difficult to manufacture, restricted in length and fragile in handling. We have instead applied inexpensive and flexible FEP tubing that was wrapped tightly around a Pyrex glass base and placed this simple unit inside a common Rayonet chamber reactor (‘outside-in’ irradiation). A different immersion well type FEP-capillary setup (‘inside-out’ irradiation) was recently reported but required a custom-built quartz tube [34]. Capillary-based reactors were originally developed for post-column photochemical derivatizations to enhance detection in HPLC [35–37] but are now commonly used in flow photochemical studies [5–9]. FEP is transparent above 230 nm and shows a good UV-stability [37].

**Figure 3:**
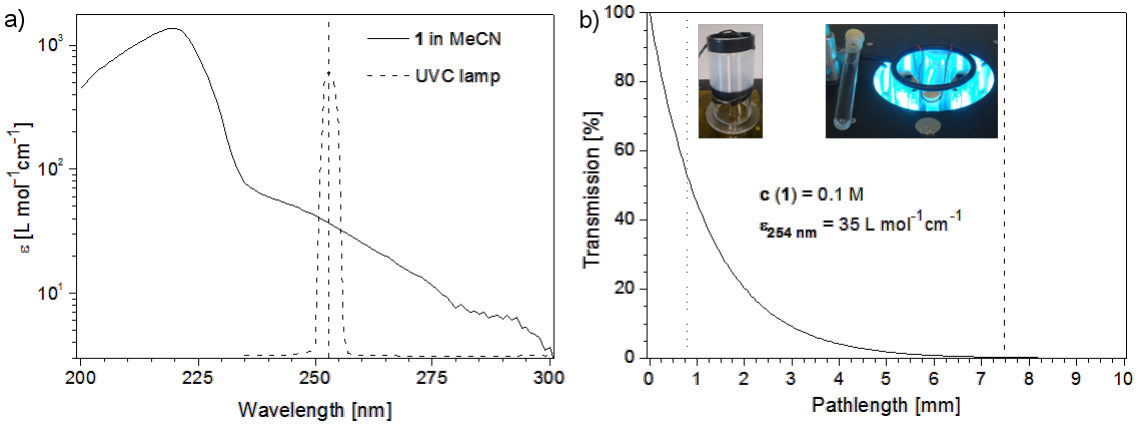
(a) UV-spectrum of **1** (in MeCN) vs emission spectrum of the UVC lamp. (b) Light-penetration profile for a 0.1 M solution of **1** at 254 nm. The vertical lines represent the effective pathlength in the test tube (---) vs the pathlength in the microcapillary (···).

In acetonitrile, furanone **1** gave a simple UV-spectrum with the important n→π* absorption as a shoulder between 240 to 270 nm. It thus matches well with the dominant emission of the UVC lamp at 254 nm (Figure 3a). At this wavelength, **1** showed an extinction coefficient (ε_254 nm_) of 35 L mol^−1^ cm^−1^. The light transmission for a 0.1 M solution of **1** was subsequently calculated from the Beer–Lambert law and was compared to the inner diameters of the reaction vessels (Figure 3b) [38]. Due to the circular arrangement of the fluorescent tubes in the chamber and hence irradiation from all directions, the effective pathlength of the test tube was reduced to 7.5 mm. Since the Pyrex base of the microcapillary module absorbed all UVC light, the microcapillary received light only from the outer direction. Due to its much smaller diameter, the light transmission in the microcapillary was still superior with 53%, compared to 0.3% in the test tube.

### [2 + 2]-Cycloadditions with cyclopentene

**Scheme 2:**
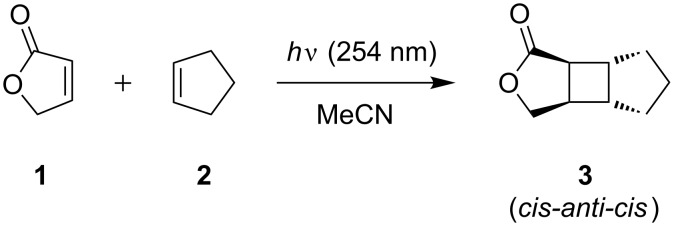
[2 + 2]-Cycloadditions of furanone **1** with cyclopentene (**2**).

The photoaddition of cyclopentene (**2**) to **1** was subsequently investigated in detail under batch and microflow conditions (Scheme 2, Table 1). Irradiation in a quartz test tube required exhaustive irradiation for 5 h to reach near completion (Table 1, entry 5) as confirmed by ^1^H NMR spectroscopy. Product isolation was performed for two batches and gave similar yields based on conversion for the *cis-anti-cis* isomer of **3** of 75% and 71% (Table 1, entries 3 and 5), respectively. In CDCl_3_, the CH_2_O-group showed a pair of doublets of doublets at 4.32 and 4.40 ppm with a ^2^*J* coupling constant of 9.5 Hz. Since the dihedral angles to the adjacent methine proton differ significantly, their ^3^*J* coupling constants varied with 2.1 and 7.3 Hz, respectively. The cyclobutane methine protons emerged as clearly separated signals between 2.35 and 2.90 ppm. Their ^3^*J* coupling constants were determined to be 2.9/3.6 and 6.7/7.5 Hz, thus confirming the *cis-anti-cis* geometry of **3**. Under continuous flow conditions, conversion rates increased more rapidly despite irradiation from just one direction. After 60 min of irradiation, 96% of furanone **1** was consumed and complete conversion was effectively achieved after 90 min (Table 1, entries 11–13). Repetition experiments were conducted with residence times of 7.5, 15 and 90 min and showed excellent reproducibility (Table 1, entries 6/7, 8/9 and 12/13). Product **3** was isolated from two experimental runs. Compared to their batch counterparts, yields based on conversion of **1** were somewhat lower with 65% and 66% (Table 1, entries 10 and 12), which was attributed to the difficult handling of the syringe pump used. The isolation of product **3** by column chromatography was also challenging as fractions had to be analyzed by material-consuming NMR spectroscopy.

**Table 1:** Experimental results for the cycloaddition of **1** with **2**.

Entry	Reactor	Time [min]	Conversion [%]^a^

1	Batch	60	28
2		90	53
3		180	71 (53^b^/75^c^)
4		240	81
5		300	95 (67^b^/71^c^)
6	μ-Reactor	7.5	38
7		7.5	40
8		15	50
9		15	53
10		30	85 (55^b^/65^c^)
11		60	96
12		90	98 (65^b^/66^c^)
13		90	98
14		120	100

^a^Determined by ^1^H NMR analysis of the crude product (±2%). ^b^Isolated yield after column chromatography. ^c^Isolated yield based on conversion.

### [2 + 2]-Cycloadditions with 2,3-dimethylbut-2-ene

**Scheme 3:**
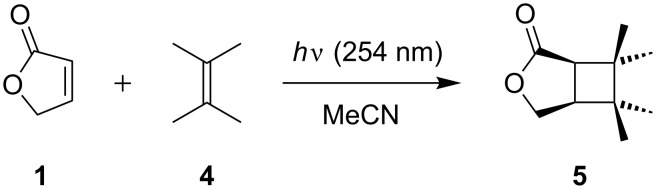
[2 + 2]-Cycloadditions of furanone **1** with 2,3-dimethylbut-2-ene (**4**).

Subsequent cycloadditions were performed using 2,3-dimethylbut-2-ene (**4**) as a reagent (Scheme 3, Table 2) [39]. In contrast to the reactions with cyclopentene, transformations were rather slow and gave more byproducts, possibly from competing ene-reactions [40]. Products arising from dimerization of **1**, however, could not be detected [41]. When conducted under batch conditions, conversions were determined as 17% after 90 min and 99% after 8 h of irradiation (Table 2, entries 1 and 2), respectively. From the latter experiment, cyclobutane **5** was isolated in a low yield of just 30%. In CDCl_3_, the CH_3_-groups in **5** gave four singlets between 1.02–1.21 ppm. Likewise, the CH_2_O-bridge appeared at 4.25 and 4.40 ppm with a ^2^*J* coupling constant of 10.1 Hz. The methine protons of the cyclobutane ring gave closely spaced signals at 2.69 and 2.73 ppm. The transformation was again more efficient under microflow conditions and conversions gradually improved with increasing retention time. Nearly complete consumption of **1** was achieved after 90 min (Table 2, entry 9). Good reproducibility was again demonstrated for reactions conducted for 30 and 60 min (Table 2, entries 5/6 and 7/8), respectively. Isolated yields based on conversion were moderate with around 45% (Table 2, entries 8 and 9).

**Table 2:** Experimental results for the cycloaddition of **1** with **4**.

Entry	Reactor	Time [min]	Conversion [%]^a^

1	Batch	90	17
2		480	99 (30^b^)
3	μ-Reactor	7.5	16
4		15	25
5		30	50
6		30	53
7		60	88
8		60	90 (41^b^/46^c^)
9		90	97 (43^b^/44^c^)

^a^Determined by ^1^H NMR analysis of the crude product (±2%). ^b^Isolated yield after column chromatography. ^c^Isolated yield based on conversion.

### Reactor comparison

Judged by conversions achieved, the microcapillary reactor showed a better performance for both [2 + 2]-photoadditions studied. This outcome is primarily attributed to the higher light and photonic efficiencies in the microcapillary, in combination with its advantageous design features and dimensions. The key parameters for both setups are compiled in Table 3. Compared to the test tube, the irradiated area-to-volume (surface-to-volume) ratio of the microcapillary module was nine times larger with 3,260 m^2^/m^3^. The microcapillary module furthermore had a better coverage of the available reflective area of the irradiation chamber, thus maximizing light harvesting by the reaction mixture. At the end of this study, the FEP microcapillary was inspected for photobrittling, transparency losses or polymeric deposits. Compared to an unused capillary, no visible or physical (e.g. flexibility) changes could be detected.

**Table 3:** Technical details of the two reactor types.

Parameter	Batch	μ-Capillary reactor

Aperture [cm^2^]	60^a^	163^b^
Irradiated area [cm^2^]	36^a^	163^b^
Irradiated volume [cm^3^]	10	5
Irradiated area/volume ratio [m^2^/m^3^]	360	3,260
Reflective chamber area/aperture	21.7/1	8.0/1
Reflective chamber area/irradiated area	36.1/1	8.0/1

^a^Assuming a cylindrical geometry for the test tube. ^b^Covered area by the microcapillary on the Pyrex base.

## Conclusion

UVC-induced photoaddition can be successfully performed in flow using a flexible and inexpensive FEP-capillary unit inserted into a common chamber photoreactor. Model transformations conducted with cyclopentene and 2,3-dimethylbut-2-ene gave higher conversions compared to the conventional quartz test tube. The microcapillary unit had a 9-times larger surface-to-volume ratio, which resulted in a more efficient harvest of the available light. The results contribute to the growing field of ‘microflow photochemistry’ [5–9] and ‘green flow chemistry’ [42–45]. It is hoped that this technology will help to overcome the current reservations towards synthetic organic photochemistry [46] and that it will find future applications in chemical and pharmaceutical processes [47–48].

## Experimental

### General

All commercially available starting materials and reagents were purchased from Sigma-Aldrich or Alfa-Aesar and were used without further purification. Furanone **1** was synthesized from furfural following literature procedures [49]. NMR spectra were recorded on an Oxford 300 (^1^H 300 MHz and ^13^C 75 MHz) with the Varian Software VnmrJ Revision D. The residual solvent signal as used as an internal standard. Chemical shifts (δ) are given in ppm; coupling constants (*J*) in Hz. IR spectra were measured on a Nicolet 6700 FTIR spectrometer equipped with a Smart ITR diamond ATR accessory. High resolution mass spectra (HRMS) were obtained on a JEOL JMS-700 instrument. Analytical thin layer chromatography was performed on Merck TLC-Silica gel 60 F_254_ plates and ethyl acetate/*n*-hexane (1:9) as mobile phase and disappearance of furanone **1** was monitored. Preparative chromatography was carried out using Scharlau silica gel 60 and ethyl acetate/*n*-hexane (1:9). Fractions taken were analyzed by ^1^H NMR spectroscopy. Irradiations were conducted in a Rayonet RPR-600 chamber reactor (Southern New England) equipped with 8 UVC lamps (4 W each). Microflow reactions were performed in a microcapillary reactor fabricated from FEP tubing (Bola, Germany; outer/inner diameter: 1.6/0.8 mm).

### Irradiations

**[2 + 2]-Cycloadditions under batch conditions:** In a quartz test tube, a solution of **1** (1 mmol) and alkene (10 mmol) in acetonitrile (10 mL) was degassed with a gentle stream of nitrogen for 5 min. The test tube was sealed and placed in the centre of a Rayonet chamber reactor. The solution was irradiated with UVC light as indicated in Table 1 and Table 2. After evaporation of the solvent, the conversion was determined by ^1^H NMR spectroscopy of the crude product. The signal integration for the olefinic CH protons of **1** was compared with the signal integration for the cyclobutane methine CH protons of **3** or **5**. In some cases, compounds **3** and **5** were isolated by column chromatography.

**[2 + 2]-Cycloadditions under microflow conditions:** A solution of **1** (1 mmol) and alkene (10 mmol) in acetonitrile (10 mL) was degassed carefully with nitrogen for 5 min and loaded into a syringe pump. The reaction mixture was pumped through the microcapillary reactor (residence times as indicated in Table 1 and Table 2) and was irradiated with UVC light. At the end of the reaction, the syringe was changed and the capillary was flushed with approx. 7.5 mL of pure acetonitrile. After evaporation of the solvent, the conversion rate was established by ^1^H NMR analysis. In selected cases, the products **3** and **5** were isolated by column chromatography.

**Octahydro-1*H*-cyclopenta[3,4]cyclobuta[1,2-*c*]furan-1-one (3)** [31]: Colorless oil; ^1^H NMR (300 MHz, CDCl_3_) δ 1.43–1.92 (br. m, 6H), 2.42 (dddd, *J* = 7.5, 7.3, 3.6, 2.1 Hz, 1H), 2.53 (dd, *J* = 7.5, 2.9 Hz, 1H), 2.65 (ddd, *J* = 6.7, 6.5, 3.6 Hz, 1H), 2.85 (ddd, *J* = 6.8, 6.7, 2.9 Hz, 1H), 4.32 (dd, *J* = 9.5, 2.1 Hz, 1H, CH_2_O), 4.40 (dd, *J* = 9.5, 7.3 Hz, 1H, CH_2_O) ppm; ^13^C NMR (75 MHz, CDCl_3_) δ 24.5, 32.7, 32.8, 37.1, 41.1, 42.6, 44.1, 74.4, 180.7 ppm; IR (ATR) ν: 2939, 2856, 1756, 1372, 1179, 1150, 1009, 980 cm^−1^; MS (EI^+^) *m*/*z*: 153 [M + H], 152 [M]^+^, 122, 93, 79, 68, 67, 53; MS (CI^+^) *m*/*z*: 305 (dimer), 193, 153 [M + H]^+^, 107, 57; HRMS (CI^+^): [M + H]^+^ calcd for C_9_H_12_O_2_, 153.0916; found, 153.0918.

**6,6,7,7-Tetramethyl-3-oxabicyclo[3.2.0]heptan-2-one (5):** Colorless oil; ^1^H NMR (300 MHz, CDCl_3_) δ 1.02 (s, 3H, CH_3_), 1.04 (s, 3H, CH_3_), 1.06 (s, 3H, CH_3_), 1.21 (s, 3H, CH_3_), 2.69 (dd, *J* = 8.4, 1.6 Hz, 1H), 2.73 (ddd, *J* = 8.4, 5.6, 1.6 Hz, 1H), 4.25 (ddd, *J* = 10.1, 5.6, 1.6 Hz, 1H, CH_2_O), 4.40 (dd, *J* = 10.1, 1.6 Hz, 1H, CH_2_O) ppm; ^13^C NMR (75 MHz, CDCl_3_) δ 20.2, 20.7, 25.9, 27.0, 39.9, 41.0, 41.3, 45.8, 68.7, 178.7 ppm; IR (ATR) ν: 2958, 2870, 1748, 1456, 1368, 1214, 971 cm^−1^; HRMS (CI^+^): [M + H]^+^ calcd for C_10_H_15_O_2_, 169.1229; found, 169.1232.
